# Awareness, knowledge, perceptions, and attitudes towards genetic testing for cancer risk among ethnic minority groups: a systematic review

**DOI:** 10.1186/s12889-017-4375-8

**Published:** 2017-05-25

**Authors:** Katie E. J. Hann, Madeleine Freeman, Lindsay Fraser, Jo Waller, Saskia C. Sanderson, Belinda Rahman, Lucy Side, Sue Gessler, Anne Lanceley, for the PROMISE study team

**Affiliations:** 1 Department of Women’s Cancer, EGA UCL Institute for Women’s Health, University College London, London, UK; 2 Department of Behavioural Science and Health, Institute of Epidemiology and Health Care, University College London, London, UK

**Keywords:** Genetic testing, Cancer risk, Ethnic minorities, Awareness, Knowledge, Attitudes

## Abstract

**Background:**

Genetic testing for risk of hereditary cancer can help patients to make important decisions about prevention or early detection. US and UK studies show that people from ethnic minority groups are less likely to receive genetic testing. It is important to understand various groups’ awareness of genetic testing and its acceptability to avoid further disparities in health care. This review aims to identify and detail awareness, knowledge, perceptions, and attitudes towards genetic counselling/testing for cancer risk prediction in ethnic minority groups.

**Methods:**

A search was carried out in PsycInfo, CINAHL, Embase and MEDLINE. Search terms referred to ethnicity, genetic testing/counselling, cancer, awareness, knowledge, attitudes, and perceptions. Quantitative and qualitative studies, written in English, and published between 2000 and 2015, were included.

**Results:**

Forty-one studies were selected for review: 39 from the US, and two from Australia. Results revealed low awareness and knowledge of genetic counselling/testing for cancer susceptibility amongst ethnic minority groups including African Americans, Asian Americans, and Hispanics. Attitudes towards genetic testing were generally positive; perceived benefits included positive implications for personal health and being able to inform family. However, negative attitudes were also evident, particularly the anticipated emotional impact of test results, and concerns about confidentiality, stigma, and discrimination. Chinese Australian groups were less studied, but of interest was a finding from qualitative research indicating that different views of who close family members are could impact on reported family history of cancer, which could in turn impact a risk assessment.

**Conclusion:**

Interventions are needed to increase awareness and knowledge of genetic testing for cancer risk and to reduce the perceived stigma and taboo surrounding the topic of cancer in ethnic minority groups. More detailed research is needed in countries other than the US and across a broader spectrum of ethnic minority groups to develop effective culturally sensitive approaches for cancer prevention.

**Electronic supplementary material:**

The online version of this article (doi:10.1186/s12889-017-4375-8) contains supplementary material, which is available to authorized users.

## Background

Several types of cancer have been found to be associated with hereditary gene mutations [1, 2]. Specifically, mutations in the *BRCA1* and *BRCA2* genes are known to be linked to breast, ovarian [3, 4], and prostate cancer [5]. Over the past 20 years, the scientific understanding of cancer related genetics has greatly improved. Within several European countries and the US, patients diagnosed with a potentially hereditary cancer or with a strong family history can receive genetic counselling and testing to establish whether they have an inherited cancer gene mutation. Knowledge about personal cancer risk can help currently healthy individuals to make health care decisions, such as whether to attend regular screening or opt for surgery, in order to help reduce the risk of developing cancer [6].

Public attitudes towards genetic testing for the risk of diseases, including cancer, have been found to be generally positive [7–9]. In a US study, 97% of participants indicated that they were at least somewhat interested in the topic of genetic testing and the majority had positive attitudes about genetic research and approved of the use of genetic testing in the detection of diseases [9]. Positive attitudes towards genetic testing are also reported in a Dutch survey study that found that 64% of participants believed genetic testing would help people to live longer [8]. However, there are also concerns amongst the general public that genetic test results could be used to discriminate against those with a genetic predisposition for illness [9] and that genetic testing could result in people being labelled as having “good” or “bad” genes [8].

The incidence and burden of cancer varies between ethnic groups. In the UK, incidence of breast cancer is higher amongst White women compared to all other ethnic groups [10], but Black men have higher rates of prostate cancer than White men [11]. Similarly, in the US overall cancer incidence and mortality has been found to be highest in Black men compared to other ethnic groups, and whilst Black women have a lower incidence of breast cancer than White women they have a worse mortality rate [12]. In the US Hispanics are reported to have lower incidence and mortality rates of cancer than White Americans [13], and evidence suggests that whilst Asian Americans also tend to have lower cancer rates than Whites, increasing incidence rates have been detected between 1990 and 2008 for breast, colorectal, and uterine cancer amongst several subgroups of Asian American women [14].

Despite the potential health benefits of cancer susceptibility testing, ethnic minority groups have been found to be underserved by genetic services and underrepresented in research in Europe and the US [15–18]. In a UK study, only 3% of patients referred to 22 regional genetics services were from an ethnic minority group [19]. Armstrong et al. [20] also found that women pursuing *BRCA1/2* genetic testing in a US study were significantly more likely to be White, and Levy et al. [21] report that significantly fewer Black and Hispanic women with a new diagnosis of breast cancer and at risk of carrying a *BRCA* gene mutation had genetic testing.

A previously published review investigating what may hinder African, White Irish, and South Asian ethnic minority groups’ access to cancer genetic services found potential barriers included low awareness and knowledge of genetic testing and available services, language barriers, stigma associated with being at risk, fatalistic views of cancer, anticipation of negative emotions, uncertainty about the information provided, and mistrust of how data would be used [22]. The current review aims to investigate factors that might act as barriers or facilitators to the uptake of genetic testing across diverse ethnic minority groups. The review fills a critical knowledge gap by focusing on awareness, knowledge, perceptions, and attitudes towards genetic testing for cancer susceptibility, and reasoning for and against testing by people from Black and ethnic minority backgrounds.

## Methods

The systematic review followed PRISMA guidelines and an a priori published protocol (https://www.crd.york.ac.uk/PROSPERO/ CRD42016033485).

### Eligibility

The review includes primary studies that aimed specifically to investigate ethnic minority groups’ awareness, knowledge, perceptions, and attitudes, or provide information on participants’ reasons for/against interest, intentions or actual uptake of genetic testing/counselling for personal cancer risk. Studies with a focus on genetic testing or genetic counselling were included as currently these services often go hand-in-hand and we wanted to see if there were important attitudes affecting uptake. We included quantitative and qualitative studies conducted in Europe, the US or Australia, and published in English in a peer reviewed journal from the year 2000 onwards.

Research that investigated awareness/attitudes/perceptions of direct-to-consumer genetic testing, or that only investigated participants’ perceptions of cancer or their experience of receiving genetic information were excluded from the review. Articles that evaluated recruitment methods, or interventions to raise awareness/knowledge of genetic testing amongst ethnic minority groups were also excluded. Studies focusing mainly on Ashkenazi Jewish participants were not included as this group is known to have an increased risk of carrying *BRCA1/2* gene mutations which might uniquely influence their knowledge/interest/attitudes to genetic testing. Ashkenazi Jewish women also have a high level of acceptance of genetic testing [23, 24]. Unpublished research was not sought for this review.

### Study search

Four databases were searched in December 2015: PsycInfo (OVID interface), CINAHL (EBSCO interface), Embase (OVID interface) and MEDLINE (OVID interface). The search terms included a combination of thesaurus (MeSH) terms and keywords such as: ‘ethnic’ or ‘minorit*’ or ‘African’ or ‘Asian’, combined with ‘know*’ or ‘aware*‘or ‘attitude*’ or ‘perception*’ and ‘cancer’ and ‘genetic testing’ or ‘genetic counselling’. [See Additional file 1 for full lists of search terms.]

### Study selection

**Fig. 1:**
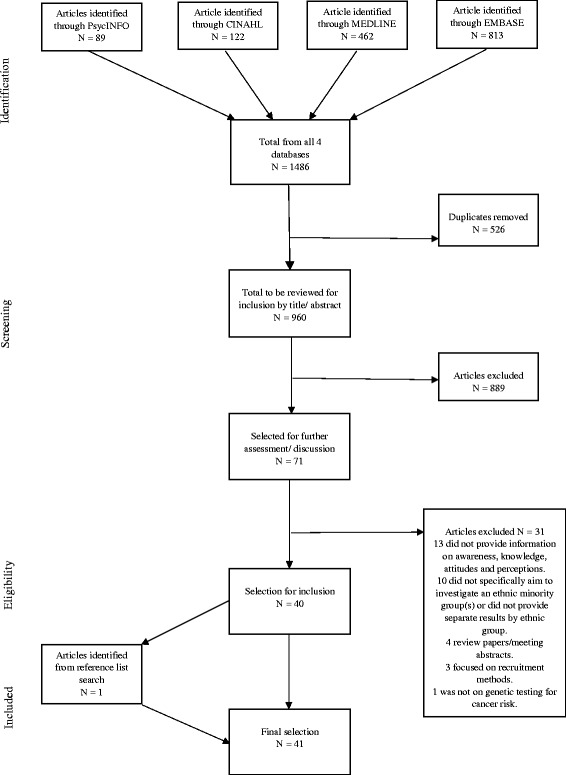
Study inclusion flow diagram. The flow diagram presents the processes of inclusion and exclusion to identify the final sample of articles to be included in the review and reasons for excluding articles

Figure 1 presents the article selection process. Study articles were independently selected by two researchers [KH & MF]. Articles were initially identified by the titles and abstracts and those which appeared eligible were reviewed in full. A reference list search was also conducted to identify additional articles. Any disagreements or uncertainties on the inclusion of studies were brought to a third researcher [AL] to make a final decision.

### Data extraction and analysis

A data extraction tool was designed to gather relevant information on the study design, methods, and results on awareness, knowledge, attitudes, and perceptions relating to genetic testing/counselling for cancer risk.

A thematic synthesis of the qualitative research was carried out in line with recommendations by Thomas and Harden [25]. The synthesis involved extracting and analysing each article’s results section, including direct quotes. An iterative process of re-reading and coding the text was used, and a coding manual was produced based on the themes identified. Once coding was completed a second researcher [MF] independently analysed 20% of the qualitative data using the coding manual. The analysis was organised using the software package NVivo (version10).

### Quality assessment

The quality of each study was assessed using tried and tested tools designed by Kmet et al. [26] for quantitative and qualitative research. The assessment tool criteria included an assessment of the description of the study objective, design, methods, data analysis, and conclusions. Criteria are scored using a 3-point Likert scale: 0 (criteria not fulfilled); 1(partial fulfilment); 2 (criteria fulfilled). A final score is calculated by adding all relevant criteria scores and dividing by the total possible score for each study. The included articles were independently quality assessed by two researchers [KH & MF]. When disagreements arose they were discussed until consensus was reached.

## Results

For the purpose of the review we have standardised the terms used to refer to the groups included, see Table 1 for a key and frequencies of groups included across the studies. Table 2 summarises the 31 quantitative studies and the results on ethnic minority groups’ awareness, knowledge, attitudes, and perceptions in regards to genetic counselling (*n* = 2), genetic testing (*n* = 27), or both (*n* = 2) for cancer risk. Measures used in the studies were often non validated study specific instruments and due to their heterogeneity across different ethnic groups and in reference to different cancers a meta-analysis of the results was not possible [see Additional file 2 for a list of the measures used]. Table 3 presents a summary of the 10 qualitative and mixed methods studies, noting themes as originally identified. Of these 10 qualitative studies, 5 involved interviews, 4 involved focus groups and 1 used both focus groups and in-depth interviews. The tables have been arranged by ethnicity and cancer type.

**Table 1:** Key for ethnicity terms

*Term used throughout*	*Includes*	*N*	*Studies*
African American	African American	23	30, 31, 32, 33, 37, 40, 42, 43, 44, 45, 46, 50, 51, 52, 53, 54, 55, 56, 57, 59, 60, 63, 65
	Black	3	29, 35, 58
	African American & West Indian	1	49
		*27*	
Hispanic	Hispanic	9	28, 30, 29, 32, 34, 36, 48, 53, 67
	Latina/o	9	27, 31, 38, 39, 40, 41, 61, 63, 66
	Puerto Rican	1	47
	Mexican/Mexican-American, Cuban-American Dominican (Republic)Central or South American		
		*19*	
Asian American	Asian American	4	31, 32, 53, 63,
White	White	5	30, 31, 32, 53, 57,
	Non-Hispanic White	1	29
	Non-Latina/o White	2	27,63
	Caucasian	4	33, 40, 42, 65
		*12*	
Other	Other	2	29, 53
Chinese Australian	Chinese Australian	2	62, 64

**Table 2:** Quantitative studies summary organised by ethnicity then cancer type

*Reference and country*	*Cancer type*	*Participants*	*Methods*	*Key findings*	*Quality assessment score*
		*African American*			
Donovan and Tucker, 2000 [42] USA	Breast	(*n* = 220) 49% African American 51% White Female 59% had a family history of breast/ovarian cancer. Average age = 41 years Average 13.5 years education Median yearly income: $25 k Groups significantly differed on age and family history of cancer.	Investigated knowledge of breast cancer and breast cancer susceptibility, and perceptions of genetic testing. Participants were recruited at a clinic and completed a questionnaire on-site. Knowledge measures were developed by combining items from previously used measures.	• White participants had significantly better knowledge of genetic risk of breast cancer (average score = 7.7 out of 14) than African Americans (average score = 7). • Breast cancer knowledge accounted for 7% of the variance in genetics knowledge and was the only significant predictor in the multivariate analysis. • African Americans were more positive about the benefits of testing (average score = 19) than Whites (average score = 18). • African Americans had more concerns about the limitations and risks of testing (average score = 13) than Whites (average score = 11).	0.95
Kinney et al. 2001 [43] USA	Breast and ovarian	(*n* = 95) African American 77% female Kindred K2099 all with increased risk of cancer based on family history. Average age = 43 years 60% had at least some college education 83% had health insurance 36% had annual household income ≥$30 k	Investigated knowledge of breast and ovarian cancer susceptibility and attitudes towards genetic testing. Known kindred participants were invited to take part by letter. The survey was completed in person (63%) or on the phone (37%). Included a knowledge measure adapted from the National Health Interview Survey.	• Participants had limited knowledge; average score of 3.2 out of 9 for knowledge about breast and ovarian cancer genetics. • Over 50% of participants agreed/strongly agreed with all (10) of the benefits of genetic testing. • Over 50% of participants agreed/strongly agreed with 5 of the 13 limitations and risks of genetic testing.	0.86
Kinney et al. 2006 [44] USA	Breast and ovarian	(*n* = 105) African American 68% female K2099 Kindred pedigree. 4.8% had a personal history of cancer. 45.6% had at least 1 first degree relative with breast/ovarian cancer. Average age < 40 years 59% had at least some college education 72.4% had health insurance 48% had annual household income ≥$41 k	Investigated predictors of *BRCA* genetic testing, including knowledge and perceptions. The survey and baseline interview took place at participants' home or other mutually agreed location. Participants were offered genetic education, counselling, and testing. Included a knowledge measure developed from Cancer Genetics Consortium survey. Also included Powe Fatalism Inventory, Religious Problem-Solving Subscales, Perceptions of Prejudice scale and other measures of cancer worry, psychological status, and social support.	• Cancer genetics knowledge was significantly higher in test acceptors (mean = 7.14) than decliners (mean = 6.11) (maximum score of 10). • Cancer genetics knowledge, perceived risk of carrying a gene mutation and age over 39 were significantly associated with genetic test acceptance. • Reasons given for not testing prior to the study: • Lack of knowledge about the test • Lack of knowledge about where to go for counselling/testing • 53% felt that their regular HCP did not have enough knowledge to provide *BRCA* related services, because they lacked training and education in genetics, or were unaware of *BRCA* or would refer on. • Cost was a barrier if they had to pay themselves: 86% would be interested if it cost $15, 49% if $150, and 15% if $1000.	0.91
Peters et al. 2004 [33] USA	Any, breast and ovarian	(*n* = 351) 48% African American 52% White 63% female General population Average age = 41 years 72% had at least some college education [significantly differed between African American (63%) and White (79%)]	Investigated awareness and knowledge of, and attitudes towards genetic testing for cancer risk. Participants recruited in a Jury selection waiting room. Questionnaires were completed on-site. Study specific questions/measures were used.	• Significantly more White (72% & 35%) than African American (49% & 25%) participants had heard of genetic testing and *BRCA* tests. • After adjusting for age, gender, and education, African Americans were significantly more likely to report that the government would use testing to label groups as inferior. • African Americans were significantly less likely to endorse potential health benefits of genetic testing, including that it could help their doctor manage their healthcare, help them change their lifestyle, help prevent cancer, or help scientists to find a cure.	0.95
Thompson et al. 2002 [45] USA	Breast and ovarian	(*n* = 76) African American Female All with at least 1 first degree relative with cancer. Average age = 43.4 years 68.4% had at least some college education 36% had annual household income ≥$40 k	Investigated predictors of *BRCA* testing uptake, including knowledge and perceptions. Part of a longitudinal study. Participants were recruited from a community cancer clinic and offered genetic counselling and testing. Included a breast cancer knowledge measure and a perceived benefits and barriers of *BRCA* testing measure developed for the study. Also included the intrusive thoughts subscale of IES.	• General breast cancer knowledge; on average participants were correct on 42.5% of the 8 questions. • Cancer genetics knowledge; on average participants were correct on 45.4% of the 14 questions. • 6/7 benefits of testing were highly endorsed (≥70%). • 7/14 barriers were endorsed by ≥50% of the women.	0.82
Edwards et al. 2008 [49] USA	Breast and ovarian	(*n* = 140) 55.7% African American 44.3% West Indian Female 69% had personally experienced breast cancer. Average age = 45.6 years 63% had at least some college education 86% had health insurance 51% had annual household income ≥$35 k	Investigated attitudes toward *BRCA* testing and association with temporal orientation. Participants were recruited by physicians and self-referral. Involved a telephone survey. A coloured answer card was posted to participants. Included a previously validated measure of temporal orientation, and a genetic testing pros and cons measure developed for the study from previous research.	• Participants highly endorsed benefits, indicating an overall positive attitude towards genetic testing. • 91–97% agreed/strongly agreed with pros relating to family. • 83–91% agreed/strongly agreed with statements indicating that test results would motivate them to carry out cancer surveillance behaviours. • 70–76% agreed/strongly agreed with positive statements on personal control. Fewer participants endorsed potential limitations of genetic testing. • 52% were concerned about the impact testing would have on their family. • 72% indicated that they would worry about other family members being carriers. • 75% indicated that they would worry about passing on the gene to their children. • Only future temporal orientation was positively associated with overall genetic testing pro score.	0.95
Hughes et al. 2003 [59] USA	Breast and ovarian	(*n* = 28) African American Female Women with 20% probability of having a *BRCA* gene mutation based on family history of cancer. Average age ≤ 50 years 61% had some college education 37% had an annual household income of >$50 k	Investigated association between cultural beliefs and uptake of genetic risk assessment and testing. Participants were recruited through mammography and oncology clinics. Offered genetic counselling and testing. Involved a telephone interview and mailed questionnaire. Included validated measures of communalism, temporal orientation, religious coping style, and cancer fatalism.	• Test acceptors scored higher (mean = 34.8 out of a possible score of 75) for fatalistic beliefs of cancer than those who declined (mean = 25.8). • No significant differences between acceptors and decliners were found on religious coping style, communalism, or temporal orientation.	0.85
Kessler et al. 2005 [50] USA	Breast and ovarian	(*n* = 74) African American Female Women with a 5–10% probability of having a *BRCA1/2* mutation. 76% had personal history of cancer. 57% had family history of cancer. Average age ≤ 50 years 72% had at least some college education 53% had an annual household income of >$35 k	Investigated attitudes towards *BRCA* genetic testing. Participants were recruited by staff at health clinics and community hospitals, health fairs, breast cancer support groups, and through self-referrals from newspaper adverts. Involved a telephone interview. Included the Powe Fatalism Inventory, adapted validated items of perceptions of cancer risk and control, previously validated measures of genetic testing attitudes and intentions.	• Participants endorsed a higher rate of benefits than limitations. Mean pro score was 18.69 and mean con score was 10.05 (out of potential 7–21). • The most important benefit was cancer prevention, which was rated as very important by 88% of participants. • The most important limitation was concern about family impact, which was rated as very important by 27% of participants. • Women diagnosed with cancer and those who perceived a higher risk were more likely to report greater cons of genetic testing.	0.91
Sussner et al. 2011 [51] USA	Breast and ovarian	(*n* = 160) African American Female 64% had personally experienced breast/ovarian cancer. 76% had a family history of cancer. Average age = 45.3 years 84% had health insurance 71% had more than a high school diploma 71% had an annual income of >$20 k	Investigated influence of ethnic, cultural and racial identity on perceptions of genetic testing for susceptibility to breast/ovarian cancer. Secondary analysis of data from a previous study in which participants were recruited through physician referral, the women’s own initiation, or community outreach. Participants were offered genetic counselling and testing. Involved a telephone interview Included the Multi-group Ethnic Identity Measure, the Multidimensional Inventory of Black Identity centrality subscale, the Africentrism scale and previously validated items measuring genetic testing pros, cons, and concerns of abuses.	• Average scores (out of 5, strongly disagree-strongly agree) indicate positive attitudes towards advantages of genetic testing: • 4.2 for pros of genetic testing • 4.4 for family related pros • Average scores (out of 5, strongly disagree-strongly agree) indicate disadvantages/risks of genetic testing are less endorsed: • 2.4 for cons of genetic testing • 3.2 for family related cons of genetic testing • 1.8 for stigma related to genetic testing • 2.2 for confidentiality concerns relating to genetic testing • 1.9 for concerns about abuses from genetic testing	0.95
Sussner et al. 2009 [56] USA	Breast	(*n* = 146) African American Female At risk of *BRCA1/2* mutation. 70% personal history of breast/ovarian cancer. 81% with a family history of breast/ovarian cancer. Average age = 45.8 years 88% had health insurance 67% had more than a high school diploma 67% had an annual income of ≥$20 k	Investigated associations between acculturation and perceptions of genetic testing. Part of a larger longitudinal study. Participants were recruited via another study and community outreach Genetic counselling was provided Involved a telephone interview. Included the Impact of Events scale, and a previously validated measure of 5 perceived barriers to genetic testing.	• Foreign-born women of African descent reported more anticipation of negative emotional reactions related to genetic testing for cancer risk than US-born women of African descent. • Women who had more avoidance symptoms for breast cancer-specific distress reported more anticipation of negative emotional reactions and more confidentiality concerns regarding genetic testing for cancer risk.	0.95
Weinrich et al. 2007 [46] USA	Prostate	(*n* = 79) African American Male All high risk 48% had personally experienced cancer. Average age = 54 years 71% had at least some college education	Investigated knowledge of hereditary prostate cancer. (Pilot study) Participants were recruited from four areas as part of a larger study. Involved a telephone interview using the Knowledge of Hereditary Prostate Cancer Scale.	• The mean knowledge score was 6.34 out of 9; this was interpreted as low knowledge of hereditary prostate cancer. • Age was a predictor of knowledge; older (>60) men had more knowledge than younger (<40) men • Education level did not predict hereditary prostate cancer knowledge.	0.80
Weinrich et al. 2002 [55] USA	Prostate	(*n* = 320) African-American Male Approximately 45% with some family history of prostate cancer. Average age = 50–59 years 54.4% had at least some college education Approximately 40.2% had an annual income ≥$25,021	Investigated predictors of intent to have a genetic prostate cancer susceptibility assessment. Participants were recruited from two research studies. Involved quantitative interviews. Used a study specific survey of intentions to test and why.	• Comments accompanying ‘yes’ responses to interest question included: • ‘It could help save my life’ • ‘If the test does not affect my health’ • ‘I would like results of test’ • ‘This is the second time I’ve had a prostate (screening test) here’ • ‘Interested in participating in all surveys’ • ‘I am glad to be part of test’ • Comments accompanying ‘no’ responses included: • ‘Genetics tests may lead to discrimination’ • ‘Too much drawing of blood; tested all the time’	0.75
Myers et al. 2000 [54] USA	Prostate	(*n* = 413) African American Male General population: 7.5% had a family history of prostate cancer. Average age ≥ 50 years 46.7% had 12 or more years of education	Investigated variables associated with intention to have a test for prostate cancer risk in the future. Involved a study specific telephone survey of items based on the Preventative Health Model and intentions to have testing.	• Weak intentions to have prostate cancer risk test were due to: • Cost (58%) • Time (52%) • Worry that others might find out about the test results (47%) • Belief that test result might be wrong (45%) • Worry that test result might indicate an increased risk (43%) • Concern about physical discomfort of testing (42%)	0.86
Satia et al. 2006 [37] USA	Colon	(*n* = 658) African Americans 57% female 6% had personally experienced cancer, 9% had a first degree relative with cancer. Average age = 43.9 years 76% had more than a high school diploma	Investigated attitudes towards genetic testing for colon cancer risk and factors associated with intentions to have testing. Participants were randomly selected from Department of Motor Vehicle rosters. Involved a mail, internet, or telephone survey. Included genetic testing familiarity, intentions, and attitudes questions adapted from previous research.	• Awareness of genetic testing: • 19.5% of participants had heard/read nothing • 33.6% had heard/read a little • 30.9% had heard/read some • 11.6% had heard/read a lot • Positive attitudes towards genetic testing for colon cancer: • 79.1% felt it would give them more control over their lives • 78.2% said it was good to know future risk of cancer • Results regarding discrimination were mixed with roughly a third agreeing (32.8%), disagreeing (31.9%) and not being sure (31.3%) that genetic testing could lead to discrimination.	1.00
McBride et al. 2005 [58] USA	Lung	(*n* = 95) 98% African American 73% female College students who were not regular smokers but had either never tried smoking or had. Average age = 19.9	Investigated beliefs about the association between genetics and lung cancer, and interest in a hypothetical test for cancer risk. (Pilot study) Involved two telephone surveys spaced five months apart. Used a study specific survey including items on use of cigarettes, susceptibility to smoking and genetic risk, and interest in genetic testing.	• Only perception of being at higher risk of lung cancer from smoking was significantly associated with interest in testing.	0.91
		*Hispanic*			
Vadaparampil et al. 2006 [34] USA	Any	(*n* = 4313) Hispanic 56.9% female General population 2.5% had personally experienced cancer. 16.5% had parents with cancer. Average age = 25–39 years 29% had at least some college education 66.6% had health insurance	Investigated awareness of genetic testing for cancer risk and the influence of acculturation. Uses data taken from the National Health Interview Survey, which used stratified sampling to oversample African Americans and Hispanics. Involed an in-person computer-assisted household interview.	• 20% had heard of genetic tests for increased cancer risk. • Awareness varied by sub-ethnic group, Puerto Ricans were most aware (27.3%) and Mexicans were least aware (14.3%). • 34.8% of those with a high level of English language preference had heard of tests, compared with 18.5% with an intermediate level and 9.5% with a low level of English language preference. • Awareness was twice as high among those born in the US than those born in another country. • Lower test awareness was associated with decreasing educational level; those who only completed high school or did not complete high school were less likely to be aware of genetic testing than those with a college education. • Test awareness was inversely associated with not engaging in physical activity and perceiving low or intermediate cancer occurrence in the family.	0.95
Heck et al. 2008 [28] USA	Any	(*n* = 10,883) Hispanic Male & female General population: 24.2% report family history of cancer. Average age ≥ 50 years	Investigated awareness of genetic testing for cancer risk and the influence of acculturation. Used data from the National Health Interview Survey 2000 and 2005. Interviews were carried out in participants’ homes.	• 17.9% of Hispanics indicated they had heard of genetic testing. • 7.7% of participants who only spoke Spanish were aware of genetic testing for cancer risk • Puerto Ricans and other Spanish had higher awareness than Mexicans/Mexican-Americans • Awareness was associated with greater use of English and length of stay in the US • Awareness was also associated with healthcare visit in past 12 months, greater educational attainment, and residence in the Midwest.	1.00
Sussner et al. 2009 [39] USA	Any	(*n* = 103) Hispanic Female 70% had personally experienced cancer. 81% had a family history of cancer. Average age = 45.19 years 27.2% had more than a high school diploma 79.8% had health insurance 12.9% had an annual income ≥$20 k	Investigated the influence of acculturation on attitudes, beliefs and familiarity with genetic testing for cancer risk. Secondary analysis of previously collected data. Involved an interviewer-administered survey completed at the recruitment site (70%) or via telephone (30%). Included the Group-Based Medical Mistrust Scale, and measures of acculturation, familiarity of genetic testing for cancer risk, and perceived benefits, barriers and concerns of abuses.	• Mean genetic testing awareness score was 7.4 out of 16. • With increasing acculturation Hispanics were more familiar with genetic testing for cancer risk. • Mean score for perceived benefits of genetic testing was 24.8 out of 30. • With increasing acculturation and decreasing medical mistrust, Hispanics were more likely to cite perceived benefits related to genetic testing for cancer risk. • Mean score for perceived barriers to genetic testing was 29.7 out of 55. • With decreasing acculturation and increasing medical mistrust, Hispanics were more likely to cite perceived barriers to genetic testing for cancer risk. • Mean score for concerns of abuses was 11.3 out of 25. • With increasing medical mistrust and age, Hispanics were more likely to cite concerns about abuses of genetic testing for cancer risk.	0.86
Vadaparampil et al. 2010 [48] USA	Breast and ovarian	(*n* = 53) Hispanic Female All high risk for cancer. 25% had personal experience of breast/ovarian cancer. 64.6% had a first degree relative with breast cancer. 56.8% had a first degree relative with ovarian cancer. Average age = 35–44 years 52.9% had at least some college education 57% had health insurance 52.8% had an annual income >$20 k	Investigated knowledge of hereditary breast/ovarian cancer. (Pilot study) Participants were recruited from clinics, support groups, health fairs, food pantries, using flyers and media press release etc. Interviewed in person at a venue selected by the participants. Acculturation measures taken from National Health Interview and knowledge measure developed by NCHGR Cancer Genetic Studies Consortium.	• Approximately half of the hereditary breast cancer questions were answered correctly, mean score of 5.15 out of 11. • Those with personal breast cancer had less knowledge than those without breast cancer.	0.85
Gammon et al. 2011 [27] USA	Breast and ovarian	(*n* = 147) 42.9% Hispanic 57.1% White Female All high risk or cancer survivors. 18.4% had prior hereditary breast cancer counselling and genetic testing. Average age = 54.5 years 68% had more than a high school diploma 78.5% had an annual household income ≥$30 k 95.1% had health insurance Groups significantly differed on personal history of cancer	Investigated awareness and attitudes in relation to *BRCA* genetic counselling and testing. Participants were recruited through the National Cancer Institute’s Cancer Genetics Network by mailed letters. Involved a computer-assisted telephone interview. Study specific questions/measures were used.	• 43.1% of Hispanic women and 65.2% of White women were aware of *BRCA1/2* testing. Hispanic responses to open ended questions: • Benefits of *BRCA1/2* testing: being able to tell family members such as daughters about test result (37.7%); would enhance prevention (31.1%); personal knowledge and awareness of risk (17.0%). Just 1.9% referred to making healthy lifestyle changes and 2.8% said for peace of mind. • Limitations: potential emotional distress (55.3%) and concern of opting for unnecessary treatment (10.5%). • Barriers: Fear (36.8) and cost (32.4%) were the most commonly sighted barriers to genetic testing, followed by availability (14.7%) and lack of awareness (13.2%). • Only 1 Hispanic woman sited “cultural barriers” as a barrier to genetic testing	0.95
Ramirez et al. 2006 [36] USA	Breast	(*n* = 48) Hispanic 67% female No personal history of breast cancer but had a family member with breast cancer (spouse, daughter, sister, mother, aunt, first cousin). Average age = 44 years 42% had at least some college education 81% had health insurance 56% had an annual household income >$25 k	Investigated awareness, interest, and perceptions of genetic testing for breast cancer risk. (Pilot study) Patients at cancer clinics, organisations and support groups were provided with information to pass on to family, those interested in participating contacted the researchers. Involved structured interviews conducted in English or Spanish. Used validated measures taken from previous research.	• Awareness of breast cancer genetic testing: • 56% had heard/read almost nothing • 27% had heard/read relatively little • 17% had heard/read a fair amount/a lot • Benefits were perceived as significantly greater than the risks/limitations • Perceived benefits of breast cancer genetic testing: • 66% (strongly) agreed they could plan for the future • 21% (strongly) agreed they could make decisions about getting married • 76% said learning if their children are at risk • 81% said to take to take better care of themselves • 83% said to get screening tests more often • Perceived risks/limitations: • 31% were concerned about their emotional reaction • 20% were concerned about their partner’s reaction • 31% were concerned about their family’s reaction • 15% felt unsure that the test is accurate • 26% would worry about how it would affect their insurance	0.75
Sussner et al. 2010 [38] USA	Breast and ovarian	(*n* = 15) Hispanic Female All high risk or experienced cancer. 40% had personally experienced cancer. 55.5% had a family history of cancer. Average age = 53.4 years 60% had at least a high school diploma 100% had health insurance 40% had an annual income ≥$20 k	Investigated interest and beliefs about genetic counselling for breast and ovarian cancer. (Pilot study) Participants were recruited from breast surgery clinics. Phase 1 involved telephone interviews. Used Group-Based Medical Mistrust Scale, measures of genetic counselling intentions, awareness, attitudes, and behavioural beliefs.	Phase 1 results: • The mean awareness score was 9.9 (scale 4–16). • Participants reported largely positive attitudes about genetic counselling for breast cancer risk (mean = 33.1, on 8–40 scale). • Behavioural beliefs score was high (mean = 63.5, on 15–75 scale), indicating more positive beliefs. • Benefits of *BRCA* genetic counselling: • 100% agreed/strongly agreed it would help them initiate discussions with family members about cancer. • 93.3% agreed/strongly agreed it would reduce fear and concerns about developing or having a recurrence of breast cancer for themselves. • Barriers to *BRCA* genetic counselling: • 73.3 agreed/strongly agreed genetic counselling could jeopardize health insurance. • 53.3% agreed/strongly agreed genetic counselling would cause them to worry about the cancer risk of family members. • 40% agreed/strongly agreed talking to a genetic counsellor would cause distress.	1.00
Sussner et al. 2013 [41] USA	Breast and ovarian	(*n* = 120) Hispanic Female All high risk or experienced cancer. 58.3% had personally experienced cancer. 46.7% had a family history of cancer. Average age = 47.5 49.2% had more than a high school diploma 88.7% had health insurance 56.4% had an annual household income ≥$20 k	Investigated facilitators and barriers of *BRCA* genetic counselling. Participants were recruited at a clinic. Involved a telephone interview. Included 5 measures of acculturation, the Group-based Medical Mistrust Scale, perceived risk of *BRCA1/2* mutation, awareness of genetic counselling for cancer risk and measures of attitudes and beliefs of *BRCA* genetic counselling. (Most measures previously developed by the team).	• Awareness of genetic counselling average score = 9.1 (possible range of 4–16) • Heard/read about genetic counselling for breast/ovarian cancer risk, mean = 2.1 (possible range of 1–5) • Knowledge about genetic counselling, sample mean = 43.8 (possible range of 11–55) • Competing life concerns was the strongest predictor of intentions to make a genetic counselling appointment. Other significant predictors included perceived risk of carrying a *BRCA* mutation and having a physician referral for genetic counselling	0.91
Vadaparampil et al. 2011 [47] USA	Breast and ovarian	(*n* = 45) Hispanic (Puerto Ricans from Tampa and Puerto Rico) Female High risk or experienced cancer. 44.5% had personally experienced cancer. 31.1% had a family history of breast cancer. 4.4% had a family history of ovarian cancer. Average age = 31–50 years 62.2% had at least some college education 55.6% had an annual income >$20 k	Investigated knowledge, interest and attitudes toward genetic testing for hereditary breast and ovarian cancer. (Pilot study) Participants were recruited through community-based outreach methods and distribution of flyers. The study involved in-depth face-to-face interviews and structured quantitative surveys, but this article focusses just on the quantitative survey data. Included Powe Fatalism Inventory, a Familism scale and previously validated measures of hereditary cancer knowledge and attitudes towards genetic testing.	• The average score for hereditary breast/ovarian cancer knowledge was 5.07 (out of 11). • Those with less than a high school education were significantly less knowledgeable about hereditary breast/ovarian cancer than those who graduated, and those who had had a diagnosis of breast cancer before age 50 were more knowledgeable than those without a diagnosis. • More participants reported that they strongly agreed/agreed with the positive attitudinal items (facilitators) compared to the negative attitudinal items (barriers), although this was not statistically significant. • The most commonly endorsed facilitator for genetic testing was a healthcare provider recommendation (100% Puerto Rico, 90% Tampa). • The most commonly endorsed barrier was that it might cost too much (32% Puerto Rico, 40% Tampa).	0.90
		*Multiple groups*			
Honda 2003 [29] USA	Any	(*n* = 31,886) 66% White 14% African American 16.6% Hispanic 3.5% Other 52.1% female General population: 9.8% report high risk of cancer. Average age = 30–49 years 51.5% had at least some college education 84.7% had health insurance 75.3% had a family annual income >$20 k The groups significantly differed on income, education and health insurance coverage.	Investigated awareness of genetic testing for cancer risk. Used data from the National Health Interview Survey 2000. Survey carried out in participants’ homes.	• Awareness of genetic testing for cancer risk by ethnicity: • 47.2% of White • 30.9% of African American • 19.1% of Hispanic • 30.5% of other • Awareness was significantly associated with race, education, immigration status, interpersonal exchange with a health care professional in past 12 months, and self-rated health status. • African American, Hispanic, and other ethnic groups were significantly less aware of genetic testing for cancer risk than Whites.	0.95
Huang et al. 2014 [30] USA	Any	(*n* = 3432) 84.8% White 6.9% Hispanic 8.3% African American 61% female General population of adult internet users. Average age = 50–60 years 81% had at least some college education 78.7% had an annual income ≥$35 k The groups significantly differed on age, education and income.	Investigated awareness of genetic testing for cancer risk amongst internet users. Used data taken from the National Cancer Institute’s HINTS survey. Involved a telephone or postal survey	• Significantly more White participants were aware of genetic testing for cancer risk (38.9%) than African American (29%) and Hispanic (27.9%) participants. • Difference in awareness between Whites and Hispanics was found to be explained by having heard of clinical trials and Centres of Disease Control, age, education, fatalism, information seeking, and discussing online information with a doctor. • Difference in awareness between Whites and African Americans was found to be explained by trust in information from religious organisations, having heard of clinical trials and Centres of Disease Control, education, living region, age, information seeking, fatalism. • Fatalistic beliefs - Hispanics (31.6%) were more likely to believe that “prevention of cancer is not possible” than African Americans (22.9%) and Whites (17.7%).	1.0
Pagan et al. 2009 [32] USA	Any	(*n* = 25,364) 66.3% White 17.6% Hispanic 13.0% African American 2.8% Asian 51% female General population Average age Whites ≥60 Average age others = 18–29 years	Investigated awareness of genetic testing for cancer risk. Used data taken from National Health Interview Survey 2005. Involved a computer-assisted interview at the participants’ home. Study specific questions/measures were used.	• Awareness of genetic testing for cancer risk: • 48.2% of Whites • 19.0% of Hispanics • 30.8% of African Americans • 27.7% of Asians • Difference in awareness between White and Hispanic participants was found to be explained in part by nativity/length of residency and education. • Difference in awareness between White and African American participants was found to be explained in part by education and region of the US. • Difference in awareness between White and Asian Americans was found to be explained in part by nativity/length of residency.	1.0
Thompson et al. 2003 [40] USA	Any	(*n* = 273) 42% African American 40% Hispanic 18% White Female 30% had at least one first degree relative with cancer. Average age = 46.1 years 48% had more than a high school diploma 89% had health insurance 45% had an annual household income ≥$20 k The groups significantly differed on income, education and health insurance coverage	Investigated negative perceptions of genetic testing for cancer risk, awareness of genetic testing, and medical mistrust. Participants were recruited through hospitals and community health care centres. Survey completed at the recruitment site or over the phone. Used the Group Based Medical Mistrust Scale, measures of awareness of genetic testing and concerns of abuses developed for the study, and a measure of perceived disadvantages of genetic testing for cancer risk based on previous research.	• Participants who had heard almost nothing/relatively little about genetic testing for cancer: • 50% White • 63.5% African American • 75.2% Hispanic • Hispanic women were significantly more likely to have reported a low level of awareness of genetic testing for cancer risk. • African Americans (mean = 29.2) and Hispanics (mean = 27.3) had significantly higher medical mistrust scores out of 60 than White (mean = 19.4). • After controlling for significant covariates there were no significant differences between ethnic groups on genetic testing disadvantages. • After controlling for covariates African Americans were found to more strongly agree with concerns of abuses compared to White participants. • African Americans and Hispanics were more likely to agree that testing will be used to show their ethnic group is not as good as others, to interfere with the way God intended people to be, and interfere with the natural order of life. African Americans were more likely to believe that genetic testing allows doctors to play God than White participants.	0.91
Armstrong et al. 2012 [57] USA	Any	(*n* = 337) 62.0% White 38.0% African American 63.5% female General population Average age = 50–59 years 57.6% had at least some college education 85.5% had health insurance 72.2% had an annual household income ≥$20 k The groups significantly differed on age, education and income.	Investigated interest in genetic testing for cancer risk under different hypothetical scenarios and healthcare system distrust. Participants were recruited through random digit dialling. Involved a telephone survey. Included a conjoint scenarios measure developed for the study and the Revised Measures of Health Care System Distrust Scale.	• Willingness to have genetic testing for cancer risk did not significantly differ between African Americans and White participants across several hypothetical scenarios. • Significantly more African Americans (26.6%) had high distrust of the health care system than Whites (17.2%). • African Americans had significantly higher healthcare mistrust scores on the values subscale (median = 16.4) compared to Whites (median = 15.4).	0.95
Kaplan et al. 2006 [31] USA	Breast	(*n* = 1711) 40% White 21% Asian American 19% African American 19% Hispanic Female Mammogram users with no personal history of breast cancer or abnormalities in most recent mammograms. 32.2% at high risk of cancer. Average age = 54.4 years 78.2% had college degree or higher 95.8% had health insurance The groups significantly differed on age, education, health insurance coverage.	Investigated awareness of cancer risk reduction therapies including genetic testing for breast cancer risk. Participants were recruited from mammography facilities. Involved telephone interviews using study specific questions/measures.	• Participants who had heard of genetic testing for breast cancer risk: • 59.4% of Whites • 31.0% of African Americans • 26.1% of Asian-Americans • 19.4% of Hispanics • Significantly more White participants had heard of genetic testing for breast cancer risk.	0.91
Armstrong et al. 2005 [53] USA	Breast and ovarian	(*n* = 408) Cases i.e. attended genetic counselling (*n* = 217) Controls i.e. did not attend genetic counselling (*n* = 191) 76.0% White 17.4% African American 3.2% Asian American 2.5% Other 1.0% Hispanic Female All with a first or second degree relative diagnosed with breast/ovarian cancer. Average age cases = 42.5 years, controls = 53.1 years Approximately 78.4% had at least some college education Approximately 98.3% had health insurance Approximately 66.6% had an annual household income ≥$30 k	Investigated factors associated with uptake of *BRCA* genetic counselling, including attitudes about genetic testing. Participants were recruited through billing databases. Involved a posted questionnaire. Included a previously validated measure of cancer worry and measures of cancer risk perception, attitudes towards genetic testing, primary care visits, and discussion of *BRCA1/2* testing.	• White women were significantly more likely to have attended genetic counselling for *BRCA* than African American women, even after controlling for the probability of a *BRCA1/2* mutation, socioeconomic factors, perceptions of cancer risk, attitudes, and discussion of testing with a primary care physician. • Use of genetic counselling was positively associated with probability of *BRCA1/2* mutation, younger age, higher breast cancer risk perception, higher ovarian cancer worry, and attitudes about discrimination and reassurance from testing. • 126/310 White, 16/71 African American, 7/13 Asian American, 0/4 Hispanic, and 10 ‘other’ participants attended testing • Of the 55 African Americans that did not have genetic testing: • 4.1% agreed testing could lead to discrimination. • 28.0% agreed that testing creates anxiety. • 42.0% agreed that testing provides reassurance. • 64.6% agreed that testing provides information.	0.95

**Table 3:** Qualitative and mixed method studies summary

*Reference and country*	*Cancer type*	*Participants*	*Methods*	*Key findings & themes*	*Quality assessment score*
		*Chinese Australian*			
Barlow-Stewart et al. (2006) [64] Australia	General	(*n* = 15) Chinese Australians 73.3% female 3 participants with some degree of family history of cancer. Age range = mid 20s – mid 80s 60% had a low acculturation level	Explored inheritance and kinship in relation to hereditary cancer. Ethnographic methods were used. Interviews took place at participants home in language of their choice. The interviews were recorded and a synopsis was produced for each participant.	• Patrilineal concept of kinship. • A weakness in the line. • Definitions of blood relatives and marriage between relatives. • Beliefs about hereditary cancer. • Interest in attending a familial cancer service.	0.85
Eisenbruch et al. (2004) [62] Australia	General	(*n* = 16) Chinese Australians 87.5% female All had family history of cancer. 6 personally affected by cancer. Average age = 43.4 years 87.5% had post school education	Explored beliefs about inheritance of cancer. Ethnographic methods were used. Interviews took place at participants’ location of choice and in language of their choice. The interviews were recorded and a synopsis was produced for each participant.	• Cancer - Naturalistic explanations. • Inheritance and hereditary (genetics) - Concepts of genes and inheritance. • Genetic testing. • Kinship issues and impact on eliciting family history. • Non-penetrance of mutations - concept of skipping generations. • Concept of spontaneous mutations followed by inheritance through the generations. • Hereditary cancer. • Screening and surveillance.	0.75
		*African American*			
Adams et al. (2015) [35] USA	Breast and ovarian	(*n* = 50) African American Female 6 participants were cancer survivors. Average age = 46–55 years 98% had health insurance 82% had at least some college education 86% had an annual income >$35 k	Explored women’s awareness and perceptions of *BRCA* testing. Participants were recruited in community settings. Survey/interviews completed on-site. The researcher made notes.	• *Quantitative results*: 54% had heard of genetic testing for breast and ovarian cancer risk; 12% had heard of *BRCA1/2* • *Qualitative themes*: knowledge: current and desired; perceived advantages and disadvantages; barriers and motivators to participating in genetic testing.	0.85
Ford et al. (2007) [65] USA	Breast	(*n* = 20) 65% African American 35% White All at risk for breast cancer. Average age ≤ 50 years	Investigated experiences of women who accepted genetic testing and those that did not despite being eligible. 3 focus groups conducted at a clinic: 1.African American women who had attended genetic counselling 2.African American women who had not attended genetic counselling 3.White women who had not attended genetic counselling Questions and analysis were based on the Preventative Health Model. The focus groups were recorded and transcribed.	• Perceptions of breast cancer. • Knowledge of breast cancer and screening. • Perceived susceptibility to breast cancer. • Worry about having breast cancer. • Interest in knowing genetic susceptibility. • Belief in breast cancer prevention and curability. • Belief in efficacy of detection and treatment in reducing breast cancer mortality. • Belief in self-efficacy related to behaviour. • Concern about behaviour related discomfort. • Support and influence of others. • Characteristics of health care delivery systems. • Reasons people would/would not go to genetic counselling. • Benefits of counselling.	0.85
Sheppard et al. (2014) [60] USA	Breast and ovarian	(*n* = 21) African American Female All with moderate to high risk of hereditary breast cancer. Average age = 38% 56–65 years 86% had some college education or more 95% had health insurance 90% had an annual income >$35 K	Explored awareness and factors that may influence uptake of genetic testing. Participants were recruited through community organisations, word of mouth and from previous research. Tape-recorded focus groups were carried out at a library, transcribed, and analysed.	• Motivators and barriers to genetic counselling/testing • Desired information about genetic counselling/testing • Women’s attitudes towards genetic counselling/testing • Information seeking and knowledge about genetic counselling/testing	0.95
Matthews et al. (2000) [52] USA	Breast, ovarian, colon and prostate	(*n* = 21) African American 62% female 95% had a family history of cancer. Average age = 42 years 66% had at least some college education 16% had an annual income ≥$35 k	Investigated informational needs regarding genetic testing for cancer risk. Participants were recruited through newspapers, hospitals, churches, radio, bulletins and fliers. Involved a questionnaire and focus groups.	*Quantitative results:* • Factors rated as important to participants’ decision to take part in testing (mean rating out of 5): Results will inform about their children’s risk (4.7) Results will inform whether other members of the family should be screened (4.6) Concern about how results will affect them (4.6) Concern of how results will affect family (4.5) Test will help plan for the future (4.4) Test results will be reassuring (4.2) Results might not be accurate (3.9) • Less important factors: Not believing that cancer can be prevented (3.4) Distrust, fear, or both of Hospitals or Physicians (3.3) Distrust, fear, or both of modern science or medicine (2.9) Not wanting others to know test results (2.6) Worry about losing insurance (2.6) *Qualitative themes:* • Lack of awareness • Communication about cancer • Perceptions about whether cancer is survivable • Emotional reactions to the process of genetic screening • Disillusionment with the medical and research communities • Testing procedure and printed materials	0.75
		*Hispanic*			
Sussner et al. (2015) [61] USA	Breast and ovarian	(*n* = 54) Hispanic Female All high risk based on family history of cancer. 26 had a personal history of breast/ovarian cancer. Average age = 49.9 years 48.1% had more than a high school diploma 92.9% had health insurance 40% had an annual income ≥$20 k	Explored beliefs and attitude about *BRCA* genetic counselling. Conducted focus groups (*n* = 54) and in-depth interviews (*n* = 30). Focus groups and interviews were conducted in Spanish or English. Interviews were conducted in person or over the phone. Interviews/focus groups were transcribed, translated, and analysed.	• Illness prevention • Personal and community knowledge about *BRCA* genetic counselling • Perceived benefits to *BRCA* genetic counselling • Cultural influences on genetic counselling participation • Influence of previous interactions with the healthcare system	0.95
Vadaparampil et al. (2010) [67] USA	Breast and ovarian	(*n* = 53) Hispanic Female All high risk of hereditary cancer. 20.8% had breast cancer and 3.8% had ovarian cancer <50 years old. Average age = 32.1% 35–44 years 52.9% had at least some college education 56.6% had health insurance 52.8% had an annual income >$20 k	Explored awareness and perceptions of genetic testing for hereditary breast and ovarian cancer. Recruited from community organisations and through media. In-depth interviews conducted with participants at a location of their choice and in the language of their choice. The interviews were recorded, transcribed, and analysed.	• Cancer risk factors • Knowledge of genetic testing • Concerns about genetic testing • Medical Doctor ever recommended genetic testing • Preferred type of medical doctor recommendation • Questions about genetic testing • Tell a friend about genetic testing	0.95
Kinney et al. (2010) [66] USA	Breast and ovarian	(*n* = 51) Hispanic 56.9% female 8% with personal history of cancer, 20% with family history of cancer. Average age = 42 years	Explored attitudes and informational needs relating to hereditary cancer and genetic testing. 5 focus groups conducted at a community centre 1. Women with high-school education or less (*n* = 11) 2. Women with some secondary education (*n* = 11) 3. Men with any educational background (*n* = 14) 4. Men with leadership positions in local community organizations (n 8) 5. Women with leadership positions in local community organizations (*n* = 7). Discussions were conducted by bilingual researchers, tape-recorded, and transcribed in English.	• Attitudes/beliefs about cancer. • Awareness of and attitudes toward hereditary breast and ovarian cancer and genetic testing. • Preferences regarding medical management for hereditary breast and ovarian cancer. • Barriers to early detection and risk reduction services • Communication issues and preferences.	0.90
		*Multiple groups*			
Glenn et al. (2012) [63] USA	Breast and ovarian	(*n* = 33) 51.5% Asian, 24.2% African American, 15.2% Hispanic 9.0% White Female 23 breast/ovarian cancer survivors, 10 high risk based on family history. Average age = 51.9 years 87.9% had health insurance 39.4% had a college education 33.3% had an annual income >$50 k	Explored potential barriers and facilitators to genetic testing. Participants identified through community organisations. Interviews were conducted by bilingual researchers, tape-recorded and transcribed in English.	• Beliefs about risk factors • Awareness of genetic testing. • Interest in genetic counselling/testing. • Cultural factors involved in genetic counselling/testing. • Perceived benefits of testing. • Perceived barriers to testing • Opinions about options following testing.	0.75

A total of 82,432 individuals from African American, Hispanic, Asian American, White, and Chinese Australian ethnic groups took part in the 41 studies reviewed. Thirty-nine of the forty-one studies were conducted in the US and two were conducted in Australia. Breast and ovarian cancer or *BRCA* genetic testing were most frequently referred to within the reviewed research, twelve referred to a number of cancers or cancer in general, three referred to prostate cancer, two to colon/colorectal cancer, and one to lung cancer.

### Quantitative studies

#### Awareness

**Fig. 2:**
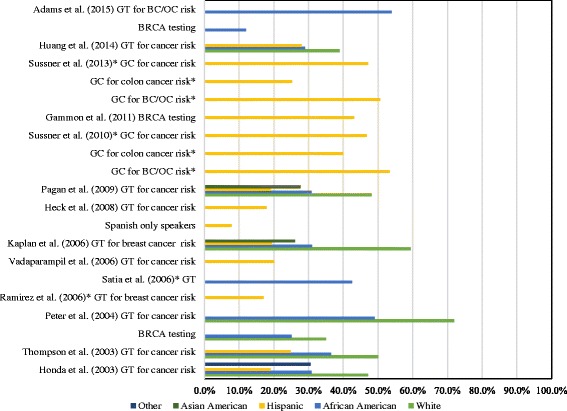
Awareness of genetic testing/counselling for cancer risk across ethnic groups. The bar graph presents the percentage of participants aware of genetic testing or counselling for cancer risk across ethnic groups. *Indicates percentage of participants that reported having read/heard a fair amount/a lot about genetic testing. BC/OC = breast/ovarian cancer; GT = genetic testing; GC = genetic counselling

Awareness was measured in two ways across 15 studies; either a dichotomous Yes/No question [27–35] or a measure asking participants how much they had heard/read about genetic testing [36–41]. Figure 2 presents the percentage of participants who indicated awareness or having heard/read a fair amount/a lot about genetic testing for cancer risk by study and ethnic group. Within general population samples of Hispanics, awareness of genetic testing for cancer risk ranged from 7.7% of Spanish only speaking Hispanics [28] to 27.9% of internet users [30]. Across two samples of Hispanics with a high risk of cancer due to family history or a personal experience of cancer, 46.7% [38] and 47.1% [41] were aware of genetic counselling for cancer risk in general; awareness of genetic counselling for colon, breast, and ovarian cancer varied. Whilst Gammon et al. [27] found 43.1% of a Hispanic sample were aware of *BRCA1/2* testing, this sample included only high cancer risk participants of whom several had already had genetic testing.

Amongst African Americans, awareness of genetic testing for cancer risk ranged from 29% [30] to 54% in general population samples [35]. Awareness of the *BRCA1/2* gene and mutation testing appears to be particularly low, one study [35] reported that only 12% were aware and another [33] reported that 25% of African American participants were aware. Two studies reported that around 25% of Asian Americans were aware of genetic testing for cancer risk [31, 32].

Five studies reported that awareness significantly differed by ethnicity, with more White participants being aware of genetic testing for cancer risk than Hispanics, African Americans, and Asian Americans [29, 30, 32, 33, 40]. Vadaparampil et al. [34] also reported that whilst 20% of their Hispanic sample were aware of genetic testing for cancer risk, awareness varied by ethnic subgroups.

Only one study assessed whether awareness was associated with intentions to have genetic counselling, and found no significant association [41].

#### Knowledge

**Fig. 3:**
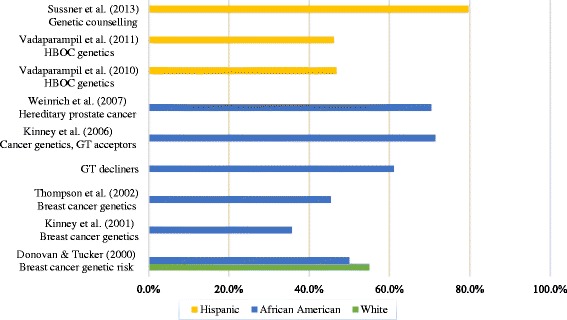
Percentage of correctly answered knowledge questions by ethnicity. The bar graph presents the average percentage of correctly answered knowledge questions across the studies and ethnic groups

Eight of the included studies measured knowledge of hereditary cancer genetics within samples at an increased risk for cancer based on family history and including individuals with a personal cancer diagnosis [41–48]. Figure 3 presents the average percentage of correctly answered knowledge questions across the studies. Findings suggest that knowledge was varied but limited amongst African Americans, with between 30% and 70% of questions answered correctly on average across five studies [42–46]. Two studies suggested that Hispanic participants also had limited knowledge, with scores of approximately 5 out of 11 for hereditary breast cancer knowledge [47, 48], although a high average score of 43.8 out of 55 was observed for genetic counselling knowledge in a third [41].

Only Donovan and Tucker [42] found a significant difference between African American and White participants’ scores on cancer genetics knowledge, indicating lower knowledge in the African American group. However the overall difference between scores was small, with a mean score of 7.7 out of 14 for Whites and 7.0 for African American.

High cancer genetics knowledge was found to be a significant predictor of genetic testing uptake in one study with African Americans [44]. In another study, those who participated in genetic counselling and testing had significantly higher scores for knowledge of cancer genetics than those who accepted neither, however cancer genetics knowledge did not reach significance as a predictor of genetic testing uptake [45]. Three other studies found no associations between cancer genetics knowledge and interest in or intentions to have genetic testing [41–43].

#### Attitudes and perceptions

Several different measures of attitudes/beliefs/perceptions regarding genetic testing or counselling were used, although similar items were used across these. Some studies reported attitude scores and others reported the number or percentage of participants who agreed with each statement. Ten studies reported that African American and Hispanic participants highly endorsed statements about the benefits of genetic counselling/testing for cancer risk, whilst endorsing limitations to a lesser extent [36–38, 41, 42, 45, 47, 49–51]. The results indicate that overall participants had positive attitudes and perceived several benefits of genetic testing.

Highly endorsed benefits of genetic counselling/testing for cancer risk included: to help make decisions on (enhanced) screening (endorsed by 81–100%) [36, 42, 43, 45, 49, 50]; to motivate self-examination (endorsed by 90–92%)[43, 45, 49]; receipt of information for family/being able to help family and children (endorsed by 38–99%) [27, 36, 38, 41, 43, 45, 47, 49, 52]; to reduce concern about cancer (endorsed by 60–90%) [38, 41, 43, 45, 49]; to reduce uncertainty (endorsed by 68–100%) [42, 43, 50]; to provide a sense of personal control (endorsed by 67–79%) [37, 43, 45, 49]; to help plan for the future (endorsed by 66%) [36, 52]. More variation was seen in attitudes towards benefits such as: to help make important life decisions (endorsed by 21–88%) [36, 41, 43, 45, 49, 50]; to provide reassurance (endorsed by 42–80% of participants) [38, 50, 52, 53]; to help with cancer prevention (endorsed by 31–88%) [27, 33, 50]; and to help make decisions about preventative surgery (endorsed by 44–77%) [41, 43, 45, 50]. Similarly, Kinney et al. [44] report that, when asked for reasons why they enrolled for genetic testing, participants cited family/personal motives (62%), information (28%), and society (9%).

Some of the most frequently endorsed limitations or barriers to genetic testing/counselling included: anticipated increased worry about offspring/relatives if test result is positive (endorsed by 53–95%) [38, 41, 43, 45, 49]; anticipated personal emotional reaction if test result is positive e.g. worry, fear, anger (endorsed 31–75%) [27, 36–38, 43, 45, 49, 52, 53]; concern about family’s reaction or impact on family (endorsed by 27–52%) [36, 49, 50, 52]; concerns about confidentiality (endorsed by 12–72%) [40, 42, 45, 49, 50]; concern about jeopardising/losing insurance (endorsed by 11–58%) [33, 36, 38, 41, 43, 45, 47, 49, 50]; cost (endorsed by 32–40%) [27, 47]; and feeling unable to handle the test emotionally (endorsed by 3–40%) [40, 42, 49, 50]. Reported reasons for a lack of interest/intention to pursue testing included cost, time, worry that others would find out, belief the results could be wrong, worry about increased risk, concern about discomfort, concern about discrimination, not wanting blood taken, logistical reasons, personal reasons, and having heard negative experiences of others who had undergone testing [44, 54, 55].

In studies comparing ethnic groups, results suggest that those from ethnic minority groups may hold less positive views or have greater concerns about genetic counselling/testing compared to White participants. Peters et al. [33] found that compared to White participants, African Americans were less likely to endorse health benefits of genetic testing and were more likely to believe that the government would use test results to label groups as inferior, however there were no other significant differences between the groups on attitudes towards insurance and job discrimination due to genetic test results. Sussner et al. [56] also reported that foreign born African American participants anticipated a more negative emotional reaction to genetic testing than those born in the US. Thompson et al. [40] found that African Americans and Latinas had significantly higher medical mistrust than Whites, and were more likely to believe that genetic test results would be used to show that their ethnic group is not as good as others, or to interfere with the natural order of things. Latinas with lower levels of acculturation and heightened medical mistrust also cited more barriers to genetic testing [39]. However, whilst Armstrong et al. [57] found that significantly more African Americans had high healthcare mistrust, there was no significant difference between African American and White participants’ willingness to undergo cancer risk genetic testing across several hypothetical scenarios.

Attitudes and perceptions were associated with uptake, interest, or intentions to have genetic testing in ten studies. Thompson et al. [45] reported that those who attended genetic counselling/testing perceived significantly fewer barriers and had more intrusive thoughts of breast cancer than those who did not. Similarly, Donovan and Tucker [42] reported that being at least somewhat interested in genetic testing was associated with more positive beliefs. Seven studies found that perceived high risk of cancer or of being a cancer gene mutation carrier was associated with acceptance of genetic testing or interest/intentions to test [41–44, 50, 53, 58]; however, one study reported that perceived cancer susceptibility was negatively associated with intentions [54]. Whilst Hughes et al. [59] found higher fatalistic beliefs about cancer in genetic test accepters than decliners, Myers et al. [54] indicated a negative association between fatalism about prostate cancer prevention and intentions to receive prostate cancer testing to find out about personal risk. Other attitudes and perceptions positively associated with genetic testing uptake or interest/intentions included the absence of the belief that testing leads to discrimination, reassurance from testing [53], belief in efficacy of screening [54], perceiving that it is good to know future risk, and believing in control through knowledge [37].

### Qualitative studies

The 10 qualitative studies included 31 Chinese Australians, 17 Asian Americans, 120 African Americans, and 163 Hispanics. The majority of participants were female and were at high risk of cancer based on family history or had a diagnosis of cancer, one study used a sample of the general population [35]. The thematic synthesis of the qualitative studies identified five broad themes: information deficits; cancer related anxiety; positive and negative attitudes and perceptions; family; and service provision and access.

#### Information deficits

Although some studies indicated that participants had good awareness and knowledge of hereditary cancer and the involvement of genetics, these participants tended to be those who had experienced cancer themselves or in a close family member [60, 61], or had previously attended genetic counselling [62, 63].

*“Genes have a big part in us having cancer. My family has a higher chance of getting cancer compared to other families as many of our family members had cancer.”*

Chinese Australian, Eisenbruch et al. [62]

Several studies including Chinese Australian, Hispanic, and African American samples reported that the majority of participants had low awareness and knowledge about the importance of genetics in cancer, *BRCA*, and genetic testing [52, 60, 61, 63–67]. Some confusion on the difference between genetic counselling and genetic testing was identified [61, 65]. Generational differences in awareness, knowledge, and beliefs were observed in studies with Hispanic and Chinese Australian samples, indicating that older generations were less aware and perhaps less open to genetic testing for cancer risk [61–64]. Some Chinese Australian participants reported that older generations and those with traditional beliefs would not accept that mutated genes can be passed through generations, attributing the development of illness instead to bad luck or past bad deeds [62, 64]. The lack of understanding about hereditary cancer and unfamiliarity with genetic testing could act as a barrier to testing. Participants across several studies expressed a need for interventions, campaigns, and the provision of information to increase awareness and knowledge of cancer and genetic testing [35, 52, 60, 61, 65].

*“Nobody knows it [genetic testing]. They are so far away [from Western medicine]. The mammogram is coming out but this is the first time I heard about the gene test.”*

Asian American female, Glenn et al. [63]

*“Black people aren’t into that yet. We need to know more about it so it will not seem so farfetched.”*

African American female, Matthews et al. [52]

Closely linked to participants’ low awareness and knowledge of cancer and genetic testing were the misconceptions held, including believing that genetic counselling prevents cancer [65], stress causes cancer [52, 63], cancer is contagious [62], injuries cause cancer [67], and focusing or worrying about cancer causes it to develop [52, 62, 64]. It is highlighted that some of these beliefs may make people less willing to discuss cancer or attend cancer services.

*“I have heard that if you hit your breasts, it could cause cancer—I saw a woman who got cancer after being hit with a bottle on her breast. My mother hit her stomach and then she got cancer in her uterus. The underwires in bras are not good and they harm your breast tissue and cause cancer. I take them out.”*

Hispanic female, Vadaparampil et al. [67]

#### Cancer related anxiety

Four studies found that African American and Hispanic participants had fatalistic views of cancer, which they perceived to be an illness that cannot be cured and is associated with death [52, 61, 65, 66]. Across all the included ethnic groups participants associated emotions such as worry, anxiety, and fear with cancer and genetic testing [35, 52, 60, 61, 63–67]. Participants expressed that fear of cancer and knowing their cancer risk was a deterrent to genetic testing [35, 52, 60, 61, 63, 65–67]. Participants anticipated experiencing anxiety whilst undergoing genetic counselling/testing [52, 64] and worried about the potential emotional impact that a positive test result would have on them [52, 67]. Some Hispanics reported that at times embarrassment prevented them from seeking medical help and attending cancer screening [63, 66]. Sheppard et al. [60] also found that women were fearful of making decisions about managing cancer risk after testing.

*“… I think it will be harder for them to find out that they have a gene or a mutation that may cause...their body to develop cancer...in the future, and so I think for a lot of people just knowing that they have the mutation will be a lot more of anxiety source than actually helpful…”*

Hispanic, Kinney et al. [66]

However, within a Hispanic community, Sussner et al. [61] found that younger generations were less fatalistic about cancer and were more enthusiastic towards genetic counselling.

*“But now the word cancer is different from before…before people were ashamed, now they believe they will beat it, it was once a taboo.”*

Hispanic female, Sussner et al. [61]

#### Positive and negative attitudes and perceptions

Interest in and positive attitudes towards genetic testing for cancer susceptibility were identified [60, 63, 66]. Personal health benefits, such as cancer prevention, early detection, and risk management, were highlighted as main benefits of genetic counselling/testing by African American, Hispanic, and Asian American participants [35, 52, 63, 65, 66]. Some participants felt that genetic testing would help to motivate them to be proactive towards their health and take action by making positive behavioural changes [35, 65] and others considered the knowledge gained from genetic testing to be empowering [61, 65].

Benefits of genetic testing included *“the opportunity for early detection, instead of waiting until the cancer develops.”*

African American female, Adams et al. [35]

Some African American and Hispanic participants discussed their spirituality and God, this was not presented as a barrier to genetic testing but as a way of seeking guidance and coping [60, 61, 67]. Some participants felt that their spirituality was a motivator for finding out about their cancer risk [65].

*“I do believe that everything is in God’s hands, regardless. But, I don’t think that my spiritual beliefs would prevent me from going. If anything, they would motivate me to go…”*

African American female, Ford et al. [65]

Several negative attitudes and perceptions were held by participants in relation to cancer and genetic testing. Discussing illnesses such as cancer was described to be taboo by Chinese Australian, African American, and Hispanic participants [61, 62, 64, 66]. Participants explained that in their culture there is secrecy around cancer and individuals might not tell family if they had it [64, 66]. This taboo is closely linked to the perceived stigma of having an illness, feeling shame and not wanting to be treated differently. Fear of discrimination by employers and insurers, and stigma was discussed across several studies and highlighted as a deterrent to testing [35, 60, 61, 63, 65, 66]. Among Asian American and Chinese Australian participants, there were also concerns that receiving a positive genetic test result would create stigma that they have “bad genes”, which could impact on marriage prospects [63, 64].

*“The stigma [would stop me from getting tested] because you always have a concern that somewhere this information is gonna reside on a computer somewhere; it may prevent you from getting employment, future insurance, or any number of things so that’s [my] concern. That is one thing that has stopped me from going ahead with testing.”*

African American female, Sheppard et al. [61]

Five of the eight qualitative studies conducted in the US reported that participants, particularly African Americans, discussed mistrust and disillusionment in the health care system [35, 52, 61, 63, 65]. Participants expressed concerns that their genetic data would be misused [63], that they would be experimented on [52], and that tests were offered to make money rather than out of necessity [52, 61]. Medical mistrust among some African Americans was reported to stem from negative events in history [52] and the “Tuskegee effect” relating to the mistreatment of African American men in the Tuskegee Syphilis study [35]. Concerns regarding confidentiality, what would happen with genetic test results after testing, and concerns about private information being discovered by others without the donor’s permission were also highlighted as important issues [52, 61, 65].

*“…I felt in the past that doctors have sent me for tests just to get money. I feel that I was put through something…really bad, going you know, put fear in me for something…just so the doctor could put the claim in and I would never trust. It was terrible…”*

Hispanic female, Sussner et al. [61]

*“One of my girlfriends who is so narrow-minded … would say ‘I wouldn’t let those people experiment [referring to genetic testing] on me’ …. Some of my other friends, who are not as narrowminded would think, ‘It’s best to find out all you can’… ‘Go for all the tests you can’ ”*

African American female, Glenn et al. [63]

Only one study, involving Asian American participants, suggested that a cultural mismatch exists between genetic testing and their culture’s traditional medicine [63]. However, scepticism of genetic testing was identified amongst Hispanic participants who were unsure of the need for genetic testing and screening without having symptoms [66], and others who felt there was no point in knowing [67]. Some African Americans were unsure of the test accuracy or reliability for their ethnic group [35, 52]. Ford et al. [65] also reported that African American women who did not attend genetic counselling perceived few benefits.

#### Family

The theme of Family was raised in discussion by many participants and was described as both a motivator and a barrier to testing. Two studies reported that Hispanics identified a culture of prioritising family needs over their own, resulting in a lack of time to attend health appointments [61, 63] and therefore a potential barrier to attending genetic counselling or testing.

*“The woman is meant to do everything, take care of the house, take care of this, take care of that, so health does go on the background…That’s how we were raised. You last, everybody else first”*

Hispanic female, Sussner et al. [61]

However, helping others in society [60, 63] and family [35, 52, 60, 61, 63, 66] was cited as an important benefit and motivator for genetic testing across most groups. Some Hispanic participants also felt it was important that they act as role models by making health more of a priority and encouraging older women to attend genetic counselling [61].

*“I was the first one diagnosed with breast cancer in my family, so I would be concerned about it for their sake to find out what was going on…so that’s what would motivate me, family.”*

African American female, Sheppard et al. [60]

Only two studies referred to family support in relation to genetic testing [61, 65]. Ford et al. [65] reported that some African American women felt they lacked family support to attend genetic counselling for cancer risk due to the potential negative impact of finding out they were high risk. Sussner et al. [61] reported that Hispanic participants felt family support would motivate them to attend genetic counselling. Both groups acknowledged that the decision was ultimately theirs; therefore, a lack of family support might not prevent individuals from having genetic counselling or testing.

*“[Family] wouldn’t keep me from going. If I wanted to go, I would go”*

African American female, Ford et al. [65]

A further issue specific to some Chinese Australians was their understanding or definition of ‘close relatives’ [62, 64]. These studies highlighted that, due to the importance placed on the paternal line, maternal family members might not always be considered ‘close relatives.’ Furthermore, when female relatives previously considered close relatives marry into other families, they may no longer be considered as such. Some indicated that relatives would be considered ‘close’ depending on how much they interacted with them rather than by bloodline [64]. These issues could lead to omissions in the assessment of cancer family history, resulting in a less accurate perception of risk.

#### Service provision and access

Several practical issues were highlighted in relation to uptake of cancer genetic testing. Ease of access to services would motivate attendance for genetic testing [60]. This could be achieved by providing services in local communities and during afternoons or weekends [35]. Physician recommendation was also an important factor in accessing services. Participants in four studies including African Americans, Hispanics, and Chinese Australians indicated that physician recommendation or referral would be a motivator to testing [35, 61, 64, 67], whilst needing yet not receiving a primary care referral would be a barrier [60, 61, 65, 67].

*“The doctor mentioned it during my last visit but he didn’t do anything about it so I didn’t do anything.”*

Hispanic female, Vadaparampil et al. [67]

As the majority of the qualitative studies took place in the US, cost and insurance were often discussed [35, 52, 60, 61, 63, 65–67]. Participants were unsure whether insurance would cover the costs of testing [67] and were unwilling or unable to pay themselves if testing was expensive or not covered by their insurance [35]. Both African Americans [65] and Hispanics voiced concerns that the cost of testing would be a particular barrier within their ethnic group due to low wages and not having health insurance [66]. Some African American participants who were affected by cancer indicated that genetic testing was too important not to have due to financial cost [60].

*“I think if I had to incur the fees myself, I wouldn’t think of going…I can’t even imagine having to pay out of my pocket for tests.”*

African American female, Ford et al. [65]

Language was identified as a barrier to using genetic services by Hispanics in two studies [61, 66]. Participants were concerned that poor communication, due also to physicians’ use of complex medical language, could have been a reason for their lack of a referral. However, some indicated that the use of a translator, including family members, had been helpful [61].

*“But if you are let’s say Spanish and you don’t know the terminology or you don’t know, they (the doctors) think the person is ignorant. That’s not the case because just because you have a language barrier does not make you ignorant.”*

Hispanic female, Sussner et al. [61]

### Quality assessment

All studies scored quite highly in the quality assessment, receiving scores of 0.75 or above (range 0.75–1.0, see Tables 1 and 2). [Additional file 3 presents the included studies in order of quality with comments on where the study failed to meet the quality criteria.] The quantitative studies scored well for sufficiently describing the study objective, design, method of subject selection, and sample characteristics. Where issues did arise, these were mainly due to shortcomings in analytic methods, for example failing to report sufficient estimates of variance, and having missing data that was not sufficiently acknowledged. Six of the included quantitative studies involved pilot research [36, 38, 46–48, 58]; however, this did not necessarily effect the study quality score, for example if it was stated that the analyses were exploratory the criteria of needing to have controlled for confounding was not applicable. The qualitative studies scored highly for sufficiently describing the study objective, design and context. However, none reflected on how the personal characteristics of the researchers may have impacted upon the analysis and results.

## Discussion

The current review supports and adds to the findings of previous work by Allford et al. [22], particularly due to the inclusion of several studies with Hispanic participants. The results highlight that only a small proportion of African American, Hispanic, and Asian ethnic minority groups are aware and knowledgeable about genetic testing for cancer susceptibility, and whilst attitudes are quite positive, concerns and negative perceptions exist. Practical issues are also highlighted as potential barriers to obtaining genetic counselling or testing.

Awareness of genetic testing for cancer risk varied across the reviewed studies; however, as recently as 2014, less than 30% of a general population sample of African Americans and Hispanics were aware [30]. The review also found some evidence that more White Americans are aware of genetic testing than Asian American, African American, and Hispanics, and this is further supported by research which did not primarily aim to investigate ethnic minority groups [68, 69]. Importantly, overall awareness of genetic testing for cancer risk was low across all ethnic groups, including White participants, highlighting that within the general population it is not a well-known health service [29, 30, 32].

Ethnic minority groups’ knowledge of genetic risk of cancer also varied across the studies reviewed, indicating low to moderate levels of knowledge. Among samples of participants at high risk for cancer, often less than 50% of knowledge questions were answered correctly. Although knowledge about cancer genetics may not relate to intentions to test, individuals who lack understanding about the hereditary nature of cancer (e.g. that *BRCA* gene mutations can be passed on by male as well as female relatives) may not pursue genetic testing that is relevant to them. Low awareness and knowledge was also highlighted in qualitative research as a barrier to attending genetic counselling or undergoing genetic testing, and participants suggested a need for awareness and educational interventions especially emphasising the importance of family history of cancer. Qualitative studies also identified several misconceptions about cancer that could deter patients from attending cancer genetic services. However, it is difficult to make generalisations based on the results from qualitative research which may only represent the views of a few individuals. Furthermore, these misconceptions do not appear to be unique to those from ethnic minority groups. For example, Ford et al. [65] report that White women who did not attend genetic counselling discussed the belief that talking about breast cancer could increase their risk; however, this misconception does not appear to have been referred to by African American women in the study. A study of White and Hispanic women found that both groups held misconceptions, including that consuming sugar substitutes and bruising from being hit can cause cancer; but such beliefs were found to be significantly more prevalent in Hispanic women [70]. Similarly, in a more recent UK study, whilst both White and South Asian participants believed that stress causes cancer, significantly more South Asians held this belief and a small minority (4.3%) also believed that cancer is contagious, a misconception that no White participants held [71].

Personal health benefits and the ability to provide information about cancer risk to the family were recognised as important benefits of genetic susceptibility testing. Family support was also discussed as a potential motivator to testing for some Hispanics, suggesting that interventions encouraging Hispanics to attend cancer genetic services could benefit from involving family. Finally, easy access to genetic services is important to facilitate uptake of genetic counselling/testing, and several qualitative studies found that a physician recommendation was a motivator for testing. Physician education on the hereditary basis of some cancers and the importance of referring people with a family history of cancer for genetic counselling is also important to ensure thorough personal risk assessment and that all patients who could benefit from testing are offered this.

Several negative attitudes and perceptions that might influence uptake of genetic susceptibility testing were found to exist among ethnic minority groups, including reluctance to talk about cancer amongst family, concerns about stigma, and concerns about the emotional reaction of undergoing genetic testing and receiving a high risk result. Whilst cancer stigma is not unique to ethnic minorities [72], it is unclear to what extent such perceived stigma and secrecy varies across different ethnic groups. Research has also found that cancer stigma varies in magnitude depending on the type of cancer [73]. As cancer stigma not only has negative consequences for patients’ psychological well-being, and can also discourage individuals from seeking help from healthcare services [74], it seems important that efforts are made to reduce stigma to encourage individuals to attend cancer genetic services.

Other perceptions and issues identified in this review appear to be more specific to certain groups. For example, perceptions of ‘close relatives’ among Chinese Australian communities could hinder risk assessment based on descriptions of family history of cancer, and Hispanic women’s culture of prioritising family over their own health could influence their behaviours relating to genetic testing and cancer risk. These various attitudes and perceptions highlight the importance of identifying the barriers and issues that are specific to different ethnic groups and the need to tailor interventions to address them.

It is particularly important to recognise that historical experiences of ethnic minority groups in different countries may result in varied attitudes towards healthcare and services such as genetic testing. There is well documented mistrust of medical research and the healthcare system among some African Americans, influenced, for example, by the Tuskegee Syphilis Study in which African American males with Syphilis were misled by researchers and refused treatment in order to study the progression of the illness [75]. Practical issues, including cost and the need for health insurance to receive testing, and concerns about insurance discrimination as a result of testing are also more relevant to some American ethnic minorities. The Genetic Information Non-discrimination Act (GINA) was signed into US law in 2008 making it illegal for health insurers and employers to discriminate against individuals on the basis of genetic information and prohibiting insurers from requiring a person to have testing or to provide genetic information. However, the act is limited as it does not cover disability, long-term care, or life insurance. Furthermore, two surveys carried out in 2009/2010 in the US, one with women at increased risk of hereditary breast/ovarian cancer [76] and one with family physicians [77], found that over half had not been aware of GINA before taking part in the survey. Considering the lack of awareness of GINA, even amongst those that might be expected to have an interest in it, and due to the limitations of the act, it is perhaps unsurprising that concerns of discrimination by insurers continues to be cited as a limitation or barrier to genetic testing in the US. In addition, several of the studies included in this review were conducted before GINA was brought into law.

Genetic testing may identify a gene change whose association with disease is not known, a ‘variant of unknown significance’ (VUS). Unlike a pathogenic high risk gene change, the association between a VUS and increased risk of cancer is unclear, leading to issues for providers (counselling, risk management and preventative care) [78] and patients (confusion, psychological impact). It has previously been estimated that approximately 10% of Caucasians undergoing *BRCA* genetic testing receive a VUS result [79], with a much higher proportion in Hispanics (9–23%) and African Americans (16–24%) [80–82]. Different prevalence rates of VUS according to ethnic group lead to differences in the ease of interpretation of results between groups. That public databases of US gene testing will have far fewer non-Whites on them than Whites contributes to the problem of interpretation. Many VUS in non-Whites will be poorly characterised compared with Whites simply because less data is available on them. The greater likelihood of receiving an uninformative VUS result in African ethnic groups might potentially contribute to a lack of understanding about genetic testing or its perceived benefits, but our review provides no evidence to support this hypothesis.

The majority of the reviewed studies were conducted in the US which limits their generalisability to other countries, particularly in relation to levels of awareness and knowledge. Nevertheless, lack of awareness, the taboo of discussing cancer, and language difficulties, have also been reported in a UK genetics service evaluation [83] and a Genetics Alliance UK report involving interviews with individuals from ethnic minorities [84]. The evaluation of a UK pilot genetic service within a culturally diverse society by Atkin et al. [83] reports that poor communication was a barrier for both White and South Asian patients; however, communication issues were most difficult for those whose first language was not English and language support was not always viewed positively. Furthermore, some female patients reported that discussing breast cancer with male practitioners was embarrassing and even felt shameful. The article highlights the benefit of employing multilingual, culturally sensitive workers within the clinic [83]. In other research, cancer fatalism has been found to be higher amongst ethnic minority women in the UK than White women, whilst cancer fear appears to vary by ethnic group [85]. Further research into the attitudes and perceptions of ethnic minority groups in the UK and other European countries is needed in order to gain a better understanding of specific cultural barriers to cancer susceptibility testing.

The review has a number of limitations including the omission of intervention studies aiming to increase participant knowledge of cancer genetics from which we may have been able to extract baseline data. We did not include unpublished literature and our experience of using the quality assessment tool, specifically chosen for its ability to assess both qualitative and quantitative studies, indicated that it lacked a finer discriminatory capacity. The heterogeneous nature of the measures of awareness, knowledge, and attitudes used across various ethnic groups and different cancer types in the quantitative studies, made a meta-analysis impossible and interpretation of results difficult. Furthermore, the majority of included studies measuring knowledge and attitudes included only at risk patients or those already with a cancer diagnosis; therefore, results may not represent the knowledge or attitudes of the general population of ethnic minorities. Whilst still in the early stages of conception, due to an increasing understanding of cancer genetics and decreases in the cost of genetic testing, population based risk stratified interventions for cancer including genetic susceptibility testing are likely to be introduced in the future. Such interventions would provide people with information on their risk of cancer, and screening or treatment would be recommended based on their estimated level of risk. Before such interventions become a reality it is necessary to gain an understanding of the attitudes of the wider population, including those who are currently underserved such as ethnic minority groups. Meisel et al. [86] found positive attitudes towards population-based genetic testing and risk stratified screening for ovarian cancer amongst a sample of women from the UK general population. However, just 34% of the sample interviewed were non-White participants and cultural/ethnic differences in attitudes and perceptions were not discussed in detail.

## Conclusions

Widening public participation amongst ethnic minority groups in future cancer risk prediction programmes may be enabled by culturally sensitive knowledge and awareness raising interventions that decrease the stigma and taboo of cancer. For this, further international research is needed to provide clearer information of ethnic minorities’ attitudes, and information and support needs so that cancer risk prediction programmes are inclusive and effective in the prevention and early detection of cancer.

## Additional files

Additional file 1: Review search terms. Full search terms for PsycInfo, CINAHL, Embase and MEDLINE used to find studies for the review. (DOCX 18 kb)

Additional file 2: Study specific measures: Awareness, knowledge, and attitudes. The table presents descriptions of the main measures used to investigate awareness, knowledge and attitudes in relation to genetic counselling/testing. (DOCX 17 kb)

Additional file 3: Rank order of included studies based on quality assessment. The table presents the quality assessments for each study and comments on why studies were marked down. (DOCX 19 kb)

## Abbreviations

GINA: Genetic Information Non-discrimination Act
UK: United Kingdom
US: United States
VUS: Variant of unknown significance
