# Supplementary material for: Awareness, knowledge, perceptions, and attitudes towards genetic testing for cancer risk among ethnic minority groups: a systematic review
Source: BMC Public Health. 2017 May 25;17:503. doi: 10.1186/s12889-017-4375-8 (PMC5445407; doi:10.1186/s12889-017-4375-8)
Supplement: Supplementary file 1 — Review search terms. Full search terms for PsycInfo, CINAHL, Embase and MEDLINE used to find studies for the review. (DOCX 18 kb) [file 12889_2017_4375_MOESM1_ESM.docx]

**Search terms**

PsycINFO (OVID interface)

1. exp Ethnic Values/
2. exp “Racial and Ethnic Attitudes”/
3. exp “Racial and Ethnic Groups”/
4. ethnic*
5. exp minority groups/
6. minorit*
7. race*
8. racial
9. cultur*
10. exp “culture (anthropological)”/
11. underserved
12. Black
13. African
14. Caribbean
15. Asian
16. Pakistani
17. Bangladeshi
18. Indian
19. Irish
20. Chinese
21. Hispanic
22. Latin*
23. Ashkenazi Jew*
24. 1 or 2 or 3 or 4 or 5 or 6 or 7 or 8 or 9 or 10 or 11 or 12 or 13 or 14 or 15 or 16 or 17 or 18 or 19 or 20 or 21 or 22 or 23
25. exp health attitudes/
26. Perception*
27. Perceive*
28. Opinion*
29. Attitude*
30. Accept*
31. Belie*
32. Know*
33. Aware*
34. Intention*
35. Interest*
36. View*
37. 25 or 26 or 27 or 28 or 29 or 30 or 31 or 32 or 33 or 34 or 35
38. Cancer*
39. genetic testing/
40. (genetic adj3 testing)
41. (gene adj3 testing)
42. genetic counseling/
43. (genetic adj3 counsel?ing)
44. (genetic adj3 screening)
45. (BRCA* adj3 testing)
46. (BRCA* adj3 screening)
47. “genetic risk assessment”
48. “risk prediction”
49. 38 or 39 or 40 or 41 or 42 or 43 or 44 or 45 or 46 or 47
50. 24 and 36 and 37 and 48

CINAHL Plus (EBSCO interface)

1. (MH “Ethnic Groups”)
2. Ethnic*
3. (MH “Minority Groups”)
4. Minorit*
5. Race*
6. racial
7. Cultur*
8. (MH “Cultural values”)
9. (MH “Culture”)
10. Underserved
11. Black
12. African
13. Caribbean
14. Asian
15. Indian
16. Pakistani
17. Bangladeshi
18. Ashkenazi Jew*
19. Chinese
20. Hispanic
21. Latin*
22. 1 or 2 or 3 or 4 or 5 or 6 or 7 or 8 or 9 or 10 or 11 or 12 or 13 or 14 or 15 or 16 or 17
23. (MH “Attitude to Health”)
24. Attitude*
25. Opinion*
26. Perception*
27. Perceive
28. Accept*
29. Belie*
30. Intention*
31. Aware*
32. Know*
33. View*
34. Interest*
35. 19 or 20 or 21 or 22 or 23 or 24 or 25 or 26 or 27 or 28 or 29
36. Cancer*
37. (MH “Genetic Screening”)
38. Genetic W3 testing
39. Gene W3 testing
40. (MH “Genetic Counseling”)
41. Genetic W3 Counsel?ing
42. Genetic W3 Screening
43. BRCA* W3 testing
44. BRCA* W3 screening
45. “Genetic risk assessment”
46. “risk prediction*”
47. 32 or 33 or 34 or 35 or 36 or 37 or 38 or 39 or 40 or 41
48. 18 and 30 and 31 and 42

MEDLINE (OVID interface)

1. Ethnic Groups/
2. ethnic*
3. Minority Groups/
4. minorit*
5. race*
6. racial
7. cultur*
8. culture/
9. underserved
10. Black
11. African
12. Caribbean
13. Asian
14. Pakistani
15. Bangladeshi
16. Indian
17. Irish
18. Chinese
19. Hispanic
20. Latin*
21. Ashkenazi Jew*
22. 1 or 2 or 3 or 4 or 5 or 6 or 7 or 8 or 9 or 10 or 11 or 12 or 13 or 14 or 15 or 16 or 17 or 18 or 19 or 20 or 21
23. Attitude to health/
24. Perception*
25. Perceive*
26. Opinion*
27. Attitude*
28. Accept*
29. Belie*
30. Know*
31. Aware*
32. Intention*
33. View*
34. Interest*
35. 23 or 24 or 25 or 26 or 27 or 28 or 29 or 30 or 31 or 32 or 33 or 34
36. Cancer*
37. genetic testing/
38. (genetic adj3 testing)
39. (gene adj3 testing)
40. genetic counseling/
41. (genetic adj3 counsel?ing)
42. (genetic adj3 screening)
43. (BRCA* adj3 testing)
44. (BRCA* adj3 screening)
45. “genetic risk assessment”
46. “risk prediction”
47. 37 or 38 or 39 or 40 or 41 or 42 or 43 or 44 or 45 or 46
48. 22 and 35 and 36 and 47

Embase (OVID interface)

1. Ethnic group/
2. Ethnic*
3. Minority group/
4. Minorit*
5. Race*
6. Racial
7. Cultur*
8. exp cultural anthropology/
9. underserved
10. Black
11. African
12. Caribbean
13. Asian
14. Pakistani
15. Bangladeshi
16. Indian
17. Irish
18. Ashkenazi Jew*
19. Chinese
20. Hispanic
21. Latin*
22. 1 or 2 or 3 or 4 or 5 or 6 or 7 or 8 or 9 or 10 or 11 or 12 or 13 or 14 or 15 or 16 or 17 or 18 or 19 or 20 or 21
23. Attitude to health/
24. Perception*
25. Perceive*
26. Opinion*
27. Attitude*
28. Accept*
29. Belie*
30. Know*
31. Aware*
32. Intention*
33. View*
34. Interest*
35. 23 or 24 or 25 or 26 or 27 or 28 or 29 or 30 or 31 or 32 or 33
36. Cancer*
37. exp genetic screening/
38. (genetic adj3 testing)
39. (gene adj3 testing)
40. Genetic counselling/
41. (genetic adj3 counsel?ing)
42. (genetic adj3 screening)
43. (BRCA* adj3 testing)
44. (BRCA adj3 screening)
45. “genetic risk assessment”
46. “risk prediction*”
47. 35 or 36 or 37 or 38 or 39 or 40 or 41 or 42 or 43 or 44 or 45
48. 22 and 34 and 35 and 46
