# Supplementary material for: Awareness, knowledge, perceptions, and attitudes towards genetic testing for cancer risk among ethnic minority groups: a systematic review
Source: BMC Public Health. 2017 May 25;17:503. doi: 10.1186/s12889-017-4375-8 (PMC5445407; doi:10.1186/s12889-017-4375-8)
Supplement: Supplementary file 2 — Study specific measures: Awareness, knowledge, and attitudes. The table presents descriptions of the main measures used to investigate awareness, knowledge and attitudes in relation to genetic counselling/testing. (DOCX 17 kb) [file 12889_2017_4375_MOESM2_ESM.docx]

Study specific measures: Awareness, knowledge and attitudes

| ***Category*** | ***Measure*** | ***n*** | ***Studies*** |
| --- | --- | --- | --- |
| **Awareness** | Dichotomous Yes/No response question.  e.g. Have you heard of genetic testing for cancer risk? | 9 | 27, 28, 29, 30, 31, 32, 33, 34, 35 |
|  | Question on extent of awareness.  e.g. How much have you read/heard about genetic testing?  Nothing/a little/some/a lot  (2 studies also summed responses to this question across 4 topics) | 6 | 36, 37, 38, 39, 40 |
| **Knowledge** | Hereditary Breast and Ovarian Cancer genetics – 11 items | 2 | 47, 48 |
|  | Breast cancer genetics – 14 items | 2 | 42, 45 |
|  | Breast cancer genetics – 9 items | 1 | 43 |
|  | Cancer genetics – 10 items | 1 | 44 |
|  | Genetic counselling | 1 | 41 |
|  | Hereditary prostate cancer – 9 items | 1 | 46 |
| **Attitudes** | Perceived barriers of genetic testing: negative emotional reaction, stigma, confidentiality, family related cons (8/10/11/13/14 items) | 7 | 39 (11 items), 40 (8 items), 43 (13 items), 45 (14 items), 49 (14 items), 51 (14 items), 56 (13 items) |
|  | Perceptions of benefits genetic testing: surveillance behaviours, personal control, family related pros (6/7 /9/10 items) | 5 | 43 (10 items), 45 (7 items), 49 (9 items), 51 (9 items), 56 (6 items) |
|  | Concerns about abuses of genetic testing (5 items) | 3 | 39, 51, 40 |
|  | Perceptions of benefits, risks and limitations of genetic testing  (14 items) | 2 | 42, 50 |
|  | Attitudes about genetic testing risks and benefits - 4 domains: information, discrimination, reassurance, anxiety (13 items) | 1 | 53 |
|  | Factors affecting decisions to participate in counselling and genetic testing (12 items) | 1 | 52 |
|  | Attitudes toward predictive genetic testing (9 items, developed from focus groups) | 1 | 33 |
|  | Perceived benefits, risks and limitations of genetic testing  (10 items, 5 pro, 5 cons) | 1 | 36 |
|  | Attitude towards genetic testing  (8 items, 3 pros, 5 cons) | 1 | 47 |
|  | Perceived benefits and risks, anxiety and confidentiality of genetic testing for colon cancer (11 items) | 1 | 37 |
|  | Attitudes about BRCA counselling  (8 items) | 2 | 38, 41 |
|  | Beliefs about BRCA counselling - 5 subcategories: pros, cons, competing life concerns, cultural concerns, logistic concerns. (26 items) | 1 | 41 |
|  | Behavioural beliefs about BRCA genetic counselling (15 items) | 1 | 38 |
