# Supplementary material for: Awareness, knowledge, perceptions, and attitudes towards genetic testing for cancer risk among ethnic minority groups: a systematic review
Source: BMC Public Health. 2017 May 25;17:503. doi: 10.1186/s12889-017-4375-8 (PMC5445407; doi:10.1186/s12889-017-4375-8)
Supplement: Supplementary file 3 — Rank order of included studies based on quality assessment. The table presents the quality assessments for each study and comments on why studies were marked down. (DOCX 19 kb) [file 12889_2017_4375_MOESM3_ESM.docx]

Rank order of included studies based on quality assessment

| ***Reference*** | ***Score*** | ***Comments*** |
| --- | --- | --- |
| ***Quantitative*** |  |  |
| Heck et al. (2008)  [28} | 1 |  |
| Huang et al. (2014)  [30] | 1 | Additional comment: Article investigates awareness of genetic testing for cancer risk but ask participants if they have ‘heard/read about *any* genetic tests’. |
| Pagan et al. (2009)  [32] | 1 | Additional comment: Older (over 60) white participants were over-represented, there were fewer older adults of other ethnicities. |
| Satia et al. (2006)  [37] | 1 |  |
| Sussner et al. (2010)  [38] | 1 | Several of the quality assessment criteria were not applicable due to explorative nature of the study and no statistical comparisons were made.  Additional comment: This is a pilot study. Small sample size (15), appropriate for study aims, but results may not be generalisable. |
| Armstrong et al. (2012)  [57] | 0.95 | Results are brief.  Additional comments: This study had a low response rate (35%). |
| Armstrong et al. (2005)  [53] | 0.95 | Small sample of ethnic minority participants. |
| Donovan & Tucker (2000)  [42] | 0.95 | Insufficient estimates of variance. |
| Edwards et al. (2008)  [49] | 0.95 | Does not clearly outline analyses. |
| Gammon et al. (2011)  [27] | 0.95 | Missing data is not sufficiently acknowledged (as ‘unknown’ in the table).  Additional comment: some participants had already had genetic counselling and testing. |
| Honda (2003)  [29] | 0.95 | Question response options were not always clear. |
| Peters et al. (2004)  [33] | 0.95 | Does not completely describe sample selection. |
| Sussner et al. (2011)  [51] | 0.95 | Some missing data not sufficiently acknowledged (as ‘unknown’ in the table). |
| Susner et al. (2009)  [56] | 0.95 | Some missing data is not sufficiently acknowledged (as ‘unknown’ in the table). |
| Vadaparampil et al. (2006)  [34] | 0.95 | Some missing data not sufficiently acknowledged (as ‘unknown’ in the table). |
| Kaplan et al. (2006)  [31] | 0.95 | Some missing data not acknowledged. |
| Kessler et al. (2005)  [50] | 0.91 | Very small sample. |
| Kinney et al. (2006)  [44] | 0.91 | Small sample size of test decliners, possible misclassification bias. |
| McBride et al. (2005)  [58] | 0.91 | Small sample, the analyses are not clear.  Additional comment: This is a pilot study. The sample was only students, so may not be generalisable. |
| Sussner et al. (2013)  [41] | 0.91 | Some results are not clear, some measures are not sufficiently described (knowledge). |
| Thompson et al. (2003)  [40] | 0.91 | Lack of estimates of variance. |
| Kinney et al. (2001)  [43] | 0.86 | Small sample, insufficient estimates of variance, some missing data not clearly acknowledged. |
| Myers et al. (2000)  [54] | 0.86 | Missing data (denominator constantly changes), the description of genetic testing given to participants is ambiguous as it does not clearly indicate the test is for genetic mutations. |
| Sussner et al. (2009)  [39] | 0.86 | Small sample, missing data (denominator constantly changes). |
| Hughes et al. (2003)  [59] | 0.85 | Small sample, does not appear to adjust for multiple testing. |
| Vadaparampil et al. (2011)  [47] | 0.85 | Small sample, insufficient estimates of variance.  Additional comment: This is a pilot study. |
| Vadaparampil et al. (2010)  [48] | 0.85 | Small sample, some missing data not sufficiently acknowledged, insufficient estimates of variance.  Additional comments: This is a pilot study with exploratory aims. |
| Thompson et al. (2002)  [45] | 0.82 | Very small sample, analyses not fully described. |
| Weinrich et al. (2007)  [46] | 0.80 | Small sample, does not appear to adjust for multiple testing, lack of estimates of variance. |
| Weinrich et al. (2002)  [55] | 0.75 | Does not appear to adjust for multiple testing, results lack detail, lack of estimates of variance. |
| Ramirez et al. (2006)  [36] | 0.75 | Small sample, questions and response options unclear, adjusting for multiple testing has not been mentioned.  Additional comments: This is a pilot study. |
| ***Qualitative/mixed methods*** |  |  |
| Sheppard et al. (2014)  [60] | 0.95 | Does not reflect on personal impact on analysis/results. |
| Sussner et al. (2015)  [61] | 0.95 | Does not reflect on personal impact on analysis/results. |
| Vadaparampil et al. (2010)  [67] | 0.95 | Does not reflect on personal impact on analysis/results. |
| Kinney et al. (2010)  [66] | 0.90 | Not clear why men of any education were selected when women of certain education levels were selected, does not reflect on personal impact of analysis/results. |
| Adams et al. (2015)  [35] | 0.85 | Convenience sample, analysis/coding not clear, does not reflect on personal impact on analysis/results |
| Barlow-Stewart et al. (2006)  [64] | 0.85 | Methods are not clear (e.g. questions asked and whether the recording was transcribed or just a synopsis was produced), does not reflect on personal impact on analysis/results. |
| Ford et al. (2007)  [65] | 0.85 | Does not reflect on factors affecting analysis/results, attempts to argue results are generalisable. |
| Eisenbruch et al. (2004)  [62] | 0.75 | Lacks detail on methods, analysis and how it was made rigorous (e.g. does not state questions asked or how data is coded), does not reflect on personal impact on analysis/results. |
| Glenn et al. (2012)  [63] | 0.75 | Lacks detail on methods, analysis and how it was made rigorous, does not reflect on own personal impact on analysis/results. |
| Matthews et al. (2000)  [52] | 0.75 | Lack of connection to theory/wider research, discussion topics not given, analysis not clear, does not reflect on personal impact on analysis/results. |
